# Effects of aspirin-loaded graphene oxide coating of a titanium surface on proliferation and osteogenic differentiation of MC3T3-E1 cells

**DOI:** 10.1038/s41598-018-33353-7

**Published:** 2018-10-11

**Authors:** Liping Ren, Shuang Pan, Haiqing Li, Yanping Li, Lina He, Shuang Zhang, Jingyi Che, Yumei Niu

**Affiliations:** 1 Department of Prosthodontics, The First Affiliated Hospital of Harbin Medical University, No. 143 Yiman Street, Nangang District, Harbin, 150001 China; 2 Department of Endodontics, The First Affiliated Hospital of Harbin Medical University, No. 143 Yiman Street, Nangang District, Harbin, 150001 China; 3 Department of Stomatology, Hospital of Heilongjiang Province, No. 82 Zhongshan Street, Xiangfang District, Harbin, 150036 China; 4 Oral Biomedical Research institute of Harbin Medical University, No. 143 Yiman Street, Nangang District, Harbin, 150001 China

## Abstract

Graphene oxide (GO) has attracted considerable attention for biomedical applications such as drug delivery because of its two-dimensional structure, which provides a large surface area on both sides of the nanosheet. Here, a new method for titanium (Ti) surface modification involving a GO coating and aspirin (A) loading (A/Ti-GO) was developed, and the bioactive effects on mouse osteoblastic MC3T3-E1 cells were preliminarily studied. The X-ray photoelectron spectrometry indicated new C-O-N, C-Si-O-C, and C-N=C bond formation upon GO coating. Remarkably, the torsion test results showed stable bonding between the GO coating and Ti under a torsional shear force found in clinical settings, in that, there was no tearing or falling off of GO coating from the sample surface. More importantly, through π-π stacking interactions, the release of aspirin loaded on the surface of Ti-GO could sustain for 3 days. Furthermore, the A/Ti-GO surface displayed a significantly higher proliferation rate and differentiation of MC3T3-E1 cells into osteoblasts, which was confirmed by a water-soluble tetrazolium salt-8 (WST-8) assay and alkaline phosphatase activity test. Consequently, Ti surface modification involving GO coating and aspirin loading might be a useful contribution to improve the success rate of Ti implants in patients, especially in bone conditions.

## Introduction

In recent decades, implant-supported denture has become the primary restorative procedure for total and partial edentulism because of its good stability, realistic aesthetics, comfort, and the ability to avoid natural tooth preparations. Titanium (Ti) and its alloys have been widely used for implant materials owing to their desirable properties such as high mechanical strength, light weight, and excellent biocompatibility^1^. Primary stability, which is defined as the biometric stability immediately after implant insertion, has a major impact on the long-term success of dental implants. Although many successful outcomes have been obtained, Ti implants still have potential failure risks because of the inherently bioinert nature of their surfaces, where they are easily oxidized^2^. Moreover, the stable oxide layer may result in thrombosis and may also lead to simple mechanical bonding between the surface and surrounding tissue^3^. The oral cavity is a challenging environment that is colonized by an impressive array of micro-organisms. During the early stages of implant placement, the inflammation around the implant increases because of the heat and excess pressure produced by drilling and screwing the implant into the bone, which hinders the growth of bone into the implants and results in weak bonding between the bone and implants^4–6^. In particular, the risk of failure is greatly increased for patients with systemic diseases such as diabetes^7^ and osteoporosis^8^. Many previous studies have proven that the surface properties of an implant strongly influence its biological behaviour. Thus, various surface modification strategies have been extensively employed to enhance the bioactive properties of Ti implants, such as anodization^9^, laser treatment^10^, ion implantation^11^, chemical vapour deposition^12^, a sol-gel technique^13^, and biomacromolecule films^14^. Among the surface modification strategies, the use of protein growth factors such as recombinant human bone morphogenetic protein-2 (BMP-2) and the silkworm protein, sericin, has been tested by applying the proteins onto the implant surfaces, to enhance osseointegration and osteogenesis in the peri-implant region^15,16^. Cai *et al*. reported that the use of TiO_2_ nanotubes as nanoreservoirs for loading BMP-2 positively promoted the migration, proliferation, and differentiation of mesenchymal stem cells. However, the chemical instability of the proteins before implant placement is a problem that needs overcoming^17^. As these methods only improved the biocompatibility and bone-bonding capacity of Ti and Ti alloys to a certain extent, the development of more promising implant surfaces is warranted in order to take advantage of the excellent characteristics of Ti and make the inert Ti surface biologically active and anti-inflammatory (to avoid early inflammation). As a result, faster and more efficient bone integration may be possible, especially for patients with conditions affecting their bones.

As a representative of nonsteroidal anti-inflammatory drugs and selective cyclooxygenase-2 (COX-2) inhibitors, aspirin has often been used as an analgesic to relieve initial aches from bone fracture and orthopaedic post-operative pain. Previous studies have proven that aspirin can prevent the development of deep venous thrombosis after implantation^18,19^. Furthermore, aspirin-treated bone marrow-derived mesenchymal stem cells have significantly improved immunomodulatory function, as indicated by upregulation of regulatory T cells and downregulation of Th17 cells^20^. Liu *et al*. reported that given the anti-inflammatory and chemotactic abilities of aspirin, co-administration of aspirin and allogeneic adipose-derived stem cells can partially reverse ovariectomy-induced bone loss in rats^21^. In addition, a study by Cao *et al*. showed that the systemic administration of aspirin was capable of improving bone marrow-derived mesenchymal stem cell-mediated calvarial bone regeneration in a large animal model^22^. It is not difficult to believe that aspirin could effectively stimulate bone formation when applied locally. Moreover, it is chemically stable and inexpensive. Therefore, if we could load aspirin onto a pure Ti surface using a vector, it would not only avoid systemic aspirin-related side effects but also accelerate implant osseointegration due to its anti-inflammatory action and promotion of enhanced peri-implant bone regeneration. The therapeutic efficacy of drugs often depends on the drug delivery carrier, which should ideally allow moderate doses to be loaded onto the surface and facilitate sustained drug release. Owing to the inert nature of the surface of Ti, which lacks rich reactive functional groups for immobilization of drug molecules, there is need for an effective carrier to load and retard the release of drugs (such as aspirin) from Ti.

In recent years, graphene oxide (GO) has attracted considerable interest in terms of its biomedical applications such as in cancer therapy^23^, tissue engineering^24^, and drug and gene delivery^25,26^ on account of its unique physical, chemical, and mechanical properties^27,28^. GO consists of single sheets of sp^2^-hybridized carbon (C) atoms arranged within honeycomb lattices. It is obtained by oxidation and exfoliation of graphite, which exhibits high dispersibility and hydrophilicity. Moreover, there is a large surface area (on both sides of the sheet) for the physical adsorption of nucleobases^29,30^ and aromatic compounds via surface adsorption, hydrogen bonding, and other types of interactions^31^. *In vitro* experiments have demonstrated that GO has high potential for promoting osteogenic differentiation. For example, a multi-biofunctional material named poly(ethylenimine) (PEI)-conjugated GO (GO-PEI) was synthesized using poly(acrylic acid) (PAA), and it was highly potent in inducing stem cell osteogenesis for fracture healing and tissue engineering^32^. La *et al*. found that GO can be used for the delivery of BMP-2 and substance P, and that this delivery promotes bone formation on Ti implants^33^. Moreover, regarding drug delivery, attaching richer functional groups to the base and edge of a GO sheet, such as epoxy (C-O-C), hydroxyl (OH), or carboxyl (COOH) groups, improves its chemical and biological properties, expanding the biomedical applications of graphene-based materials. Yang *et al*. developed a new drug carrier based on functionalized GO, which could achieve targeted delivery of doxorubicin into cancer cells overexpressing CD44 receptors and allow controlled release of doxorubicin at an acidic pH^34^. It was also reported that heparin-modified GO is a promising candidate as an ideal nanocarrier for drug delivery and anti-cancer therapy^35^. However, there is little research on GO as a drug carrier that can be used to modify implant materials.

In this study, a new method of Ti surface modification involving GO coating (by employing an alkali-hydrothermal reaction and a silane-coupling agent) was developed. A torque test (imitating the clinical rotating dental implantation process) was then employed to detect the bonding strength between the GO coating and Ti substrate, which has not been reported before. Subsequently, aspirin was loaded onto the surface of Ti-GO via π–π stacking, and the biological effects were preliminarily studied.

## Results

### Characterization of GO and Ti-GO

**Figure 1:**
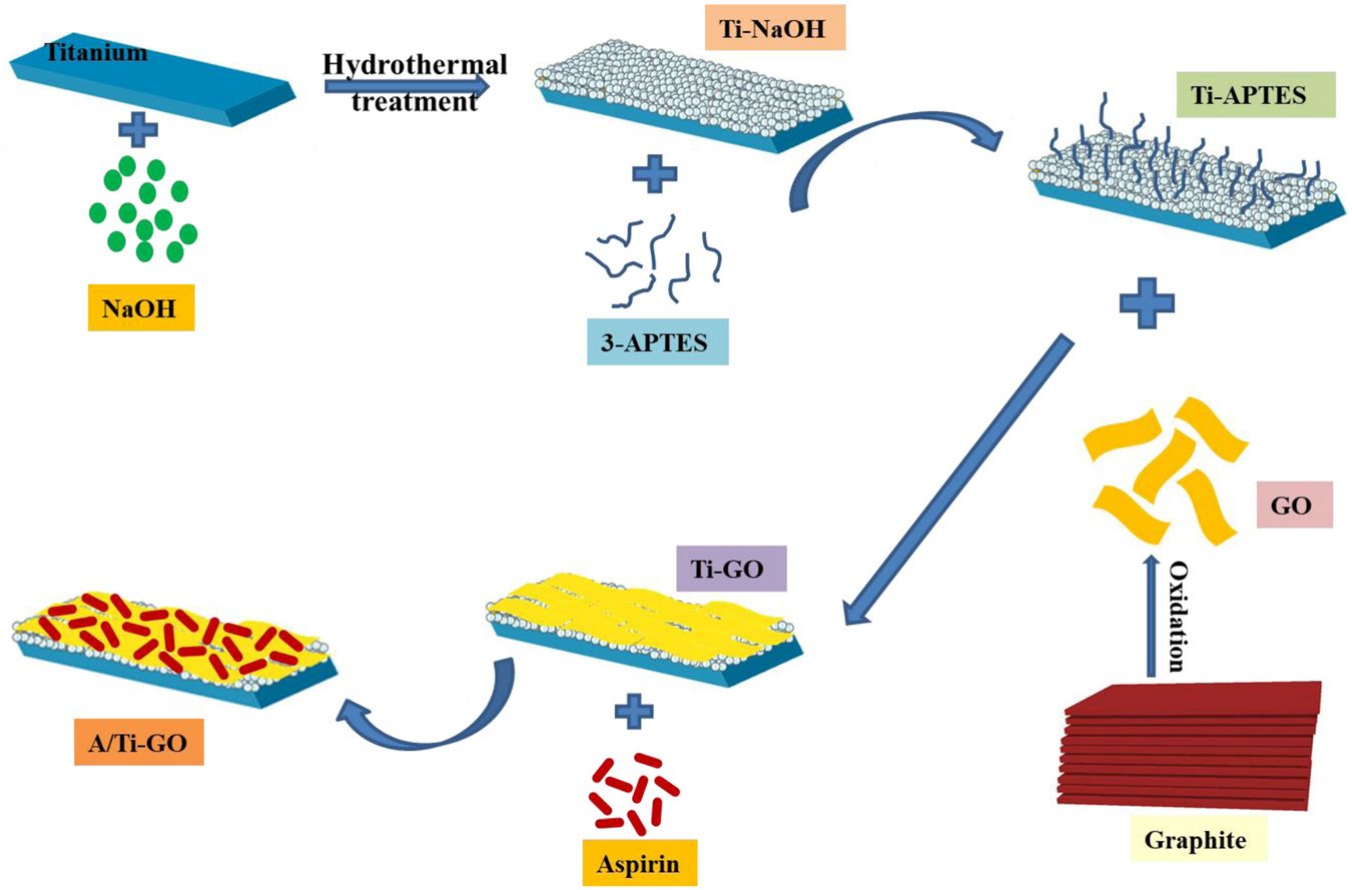
Schematic illustration of the preparation of the Ti surface modified with a GO coating and aspirin loading.

An overview of the preparation of A/Ti-GO samples is shown in Fig. 1. Firstly, GO was prepared via an improved Hummers’ method and nanonetwork-structured substrates were simultaneously prepared on Ti surfaces using an alkali-hydrothermal reaction. GO was then sequentially conjugated to the surface of the modified Ti with the aid of 3-APTES functional groups. Lastly, aspirin was loaded onto the Ti-GO surfaces via π-π stacking.

**Figure 2:**
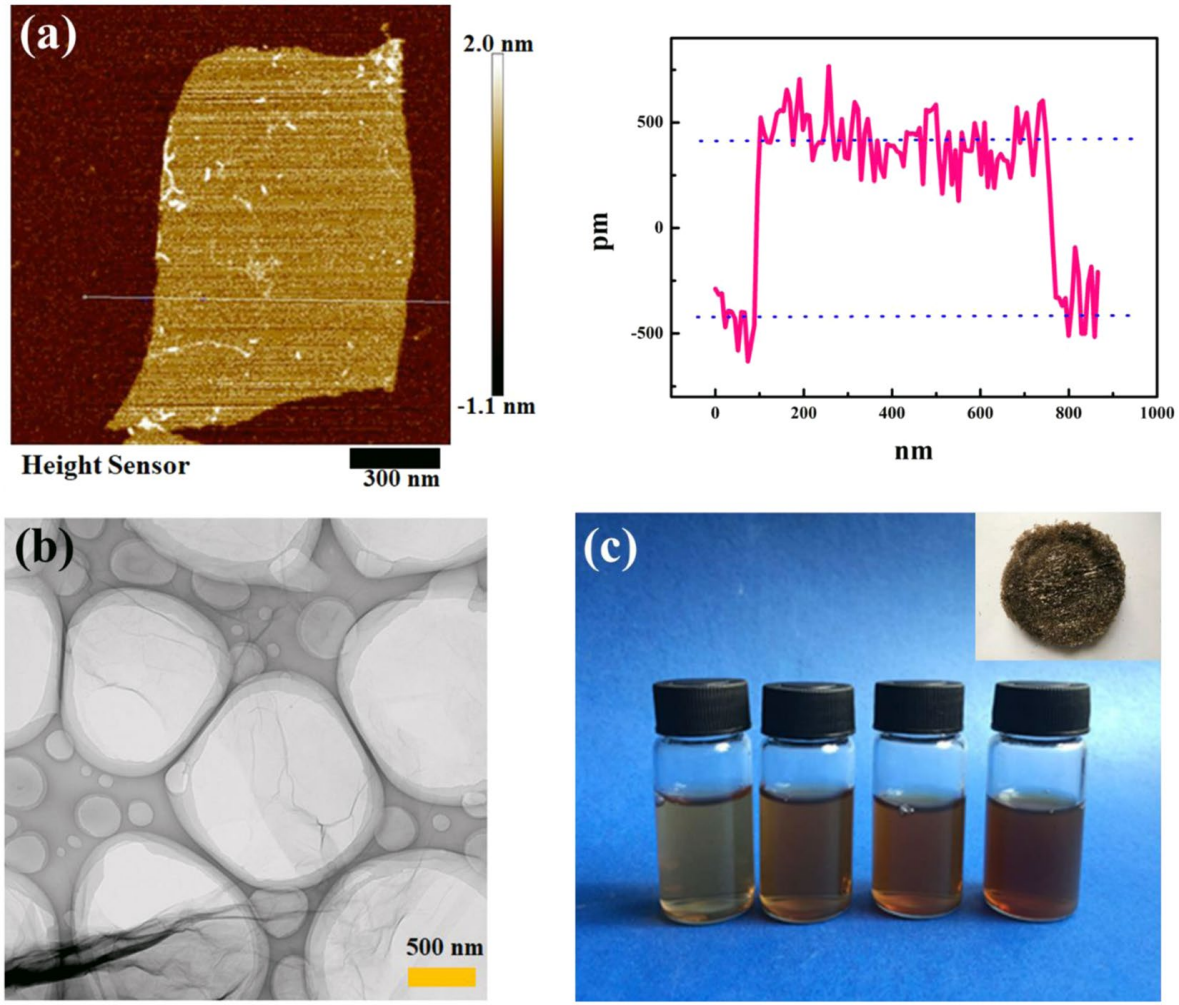
Characterization of the synthesized GO. (**a**) AFM image of single-layer graphene with a topographic height of approximately 1.29 nm. (**b**) TEM image of GO. (**c**) Photo of GO dispersed in ultrapure water: 0.5 mg/mL, 1.0 mg/mL, 1.5 mg/mL, and 2.0 mg/mL from left to right, and a photo of synthesized GO powder is shown in the inset.

The special one-dimensional nanostructure of the synthesized GO was verified beforehand. AFM demonstrated that the GO was very flat and unwrinkled on the mica surface, as shown in Fig. 2a. The sheet thickness was approximately 1.29 nm, proving that GO consists of a two-dimensional sheet of covalently bonded C atoms, which is consistent with a previous report^36^. After dripping the GO solution onto a copper net, TEM (Fig. 2b) indicated the presence of a lamellar structure deposited over many circular wells forming a series of free-standing membranes, and part of the area overlapped, resembling a thin curtain because of liquid accumulation. Further, the synthesized GO layer was shown to be large and ultrathin, which is consistent with the AFM results. Because of the presence of hydrophilic oxygen (O) functional groups on its surface and edge, the GO was easily exfoliated in aqueous media, which produced a light-yellow product. The colour deepened with an increase in concentration (Fig. 2c).

**Figure 3:**
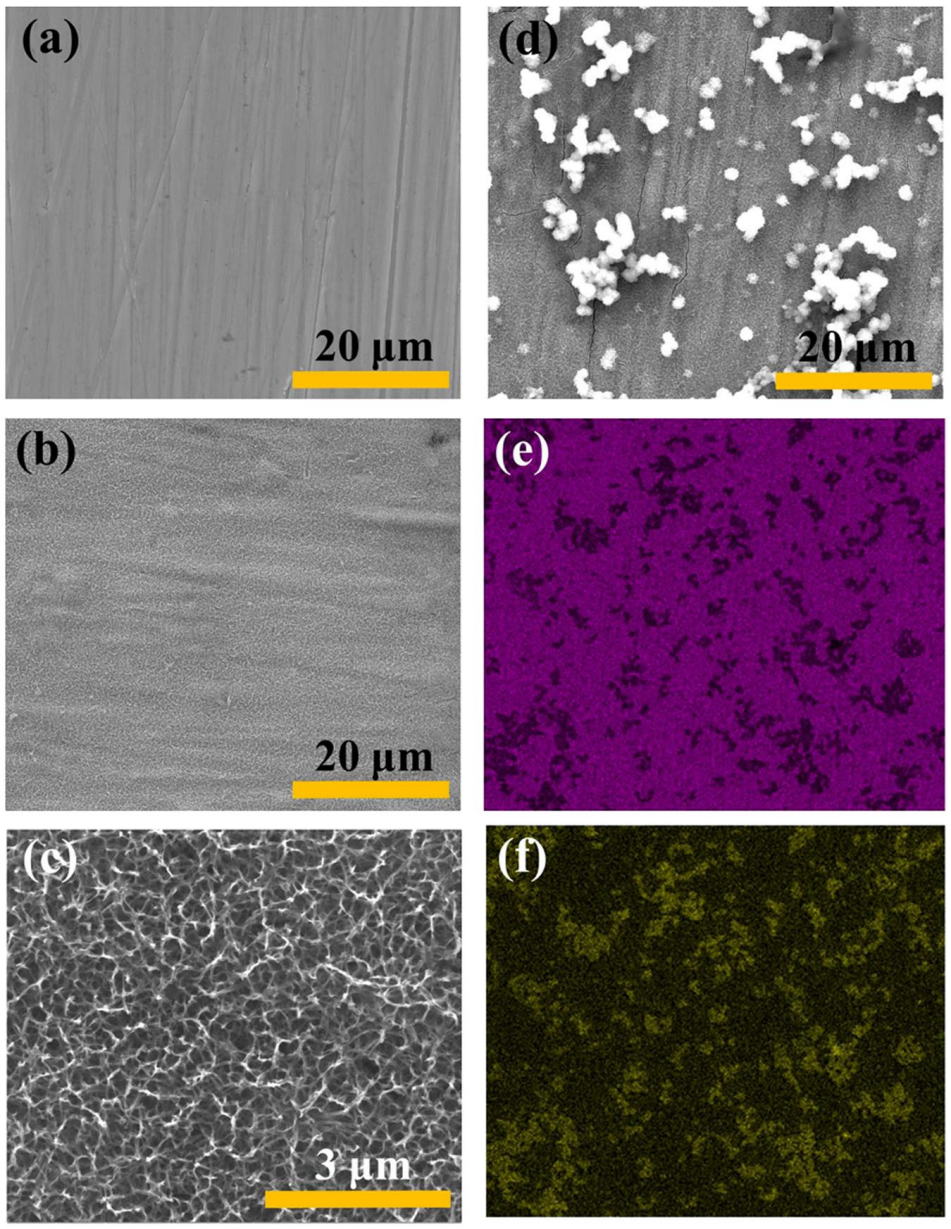
Surface characterizations of Ti, Ti-NaOH, and Ti-APTES, which are related to the preparation procedure for Ti-GO. FE-SEM: (**a**) Ti, (**b**) Ti-NaOH, (**c**) higher magnification of Ti-NaOH, (**d**) Ti-APTES. Surface distribution image: (**e**) The Ti elements are marked in purple, (**f**) The silicon elements are marked in yellow.

The surface morphologies relevant to the Ti-GO preparation procedure were characterized using FE-SEM (Fig. 3a–d). The bare Ti surface was smooth and consistent after polishing. An alkali-hydrothermal reaction was employed as an ideal approach to induce nanoscale topography on the top layers of the Ti substrates. After using the silane coupling agent, most of the silane coupling agent particles were uniformly dispersed on the surface. This introduced active functional groups to the surface in order to allow GO coating. Similarly, in the surface distribution images (Fig. 3e,f), it can be clearly seen that the Ti (marked in purple on the base surface) and the silicon (marked in yellow) elements were evenly distributed on the surface.

**Figure 4:**
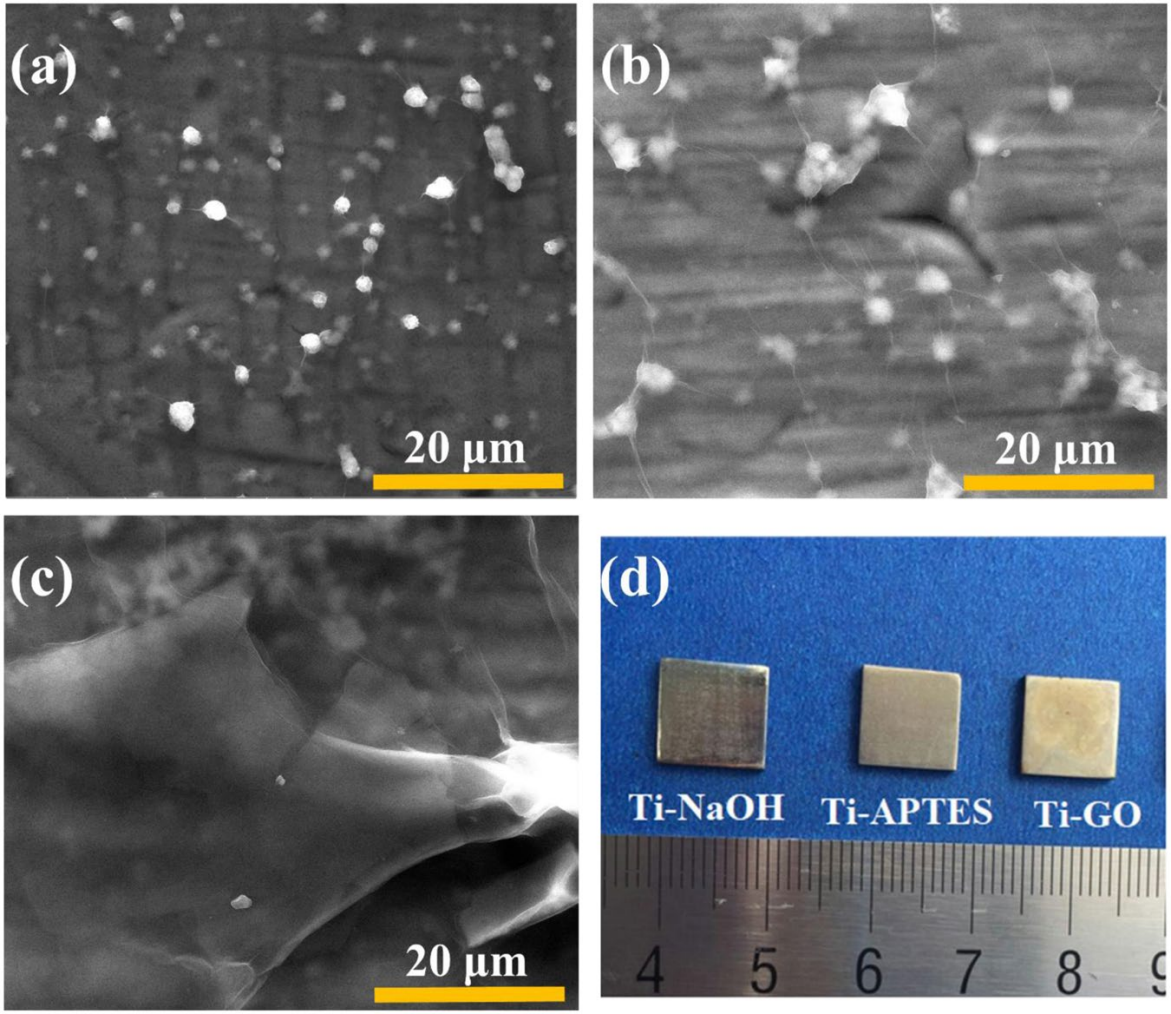
Surface characterizations of Ti-GO. FE-SEM: (**a**) 1.0 mg/mL Ti-GO, (**b**) 1.5 mg/mL Ti-GO, (**c**) 2.0 mg/mL Ti-GO. (**d**) Digital photos related to the preparation procedure for Ti-GO.

After immersion in different concentrations of GO solution (Fig. 4a–c), GO did not cover all the surface of the Ti samples in the 1.0 mg/mL group. However, GO formed a thick coating in the 2.0 mg/mL group. In comparison, the 1.5 mg/mL functionalized Ti-GO group exhibited a homogeneous morphological layer of GO films, and the granulated silane coupling agent below was clearly visible through the GO membrane. This sample group was used for the subsequent experiments. As shown in the digital photos (Fig. 4d), a light-yellow film was formed on the surface of the Ti sample after GO coating.

**Figure 5:**
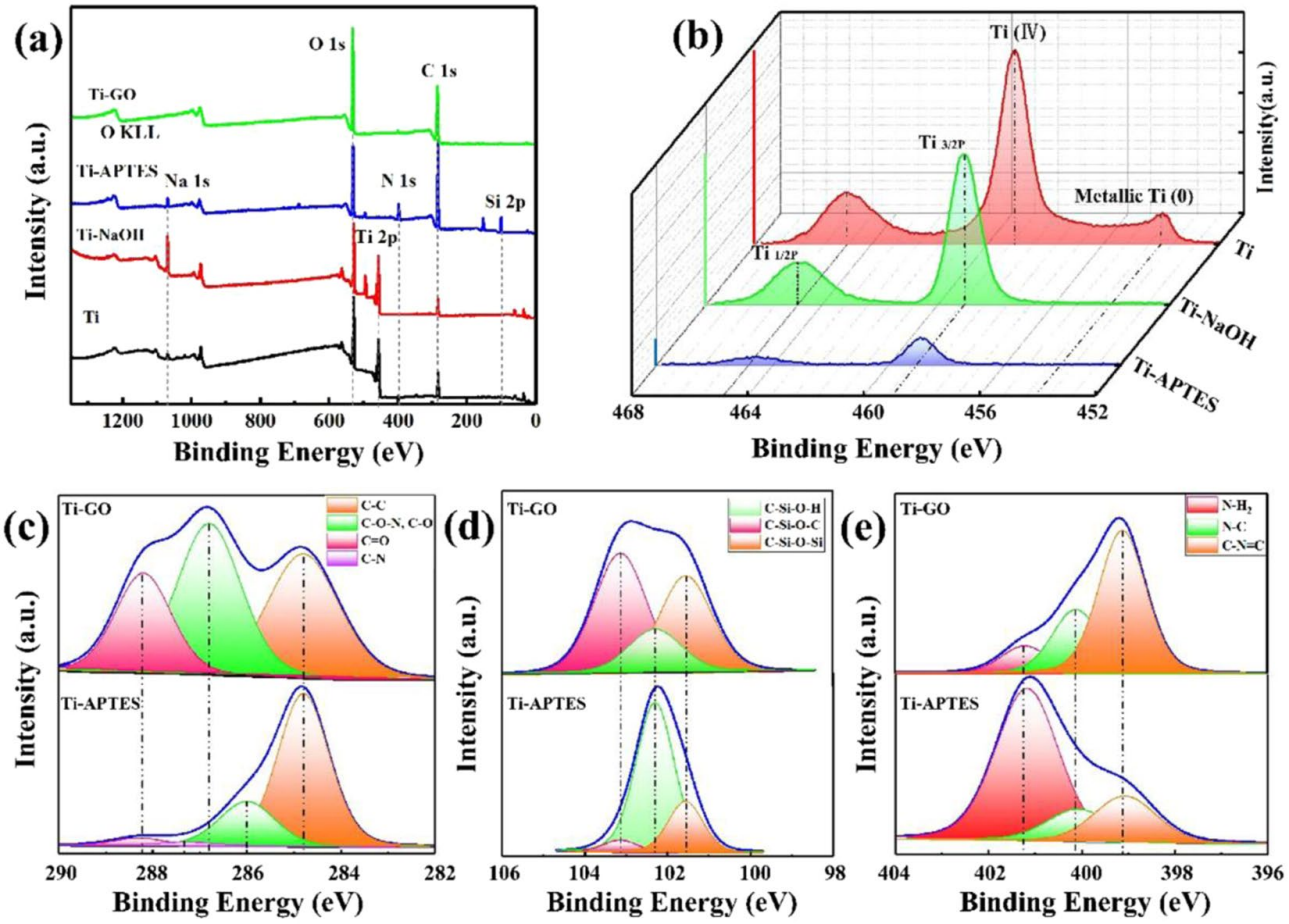
XPS spectra. (**a**) XPS survey scans and fitted curves for (**b**) Ti, (**c**) C, (**d**) Si and (**e**) N.

The characterization of the Ti-GO surface was further validated by XPS to analyse the surface elements and confirm the FE-SEM results. Figure 5 shows the XPS survey scans and the fitted curves for Ti, C, Si, and N. As shown in Fig. 5a, characteristic Na peaks were detected on the surface of Ti-NaOH. In the case of Ti-APTES, the presence of additional N and Si peaks were observed. After the formation of the Ti-GO coating, there were intense C and O peaks in comparison with the peaks for other samples due to the considerably different degree of O functional groups in the GO structure. Conversely, the intensity of Ti, Na, N, and Si peaks decreased dramatically.

As shown in Fig. 5b, the Ti XPS spectra show that Ti completely formed the high states of Ti_1/2p_ and Ti_3/2p_ under NaOH oxidation. Although XPS provides elemental and chemical state data for within 10 nm of the surface, the high state of Ti_1/2p_ and Ti_3/2p_ could still be detected on the surface of Ti-APTES as the silane coupling agent coating was very thin and uniform.

Figure 5c presents the fitted curve for C in Ti-APTES and Ti-GO. The C XPS spectrum of Ti-APTES can be deconvoluted into four peaks, which are attributable to the C-C, C-O, C-N, and C=O of the coupling agent. In the C spectrum of Ti-GO, three types of C with different chemical state locations are observed^37^, because of the changes in the surrounding chemical environments. Moreover, the relative peak areas for the O-containing groups of GO were increased significantly after the process of oxidation (of natural flake graphite), including the peaks for C-O (for the epoxy groups) and O-C=O (for the carboxyl groups), which occupy up to 63.3% of the total peak area. Hence, this further confirms that we acquired highly oxidized GO. Furthermore, we identified new chemical bonding involving C-O-N, C-Si-O-C, and C-N=C between 3-APTES and GO, as shown in Fig. 5c–e. The result confirmed that the Ti-GO coating was achieved by an alkali-hydrothermal reaction and silane coupling agent assistance.

Water contact angle measurement is a method that indicates the hydrophobic/hydrophilic properties of measured substrates. Figure S1 shows that the order of the surface water contact angles was Ti>Ti-APTES>Ti-GO>Ti-NaOH. Compared with Ti, the contact angle after GO coating was considerably decreased by 23.72° (*P* < 0.05).

### Cytotoxicity

To investigate the cytotoxicity of Ti-GO, MC3T3-E1 cells were used to perform the water-soluble tetrazolium salt-8 (WST-8) assay. As shown in Fig. S2, no significant difference in cell proliferation between Ti and Ti-GO was observed after 1 or 3 days (*P* > 0.05).

### Torsion experiment to examine bonding strength

**Figure 6:**
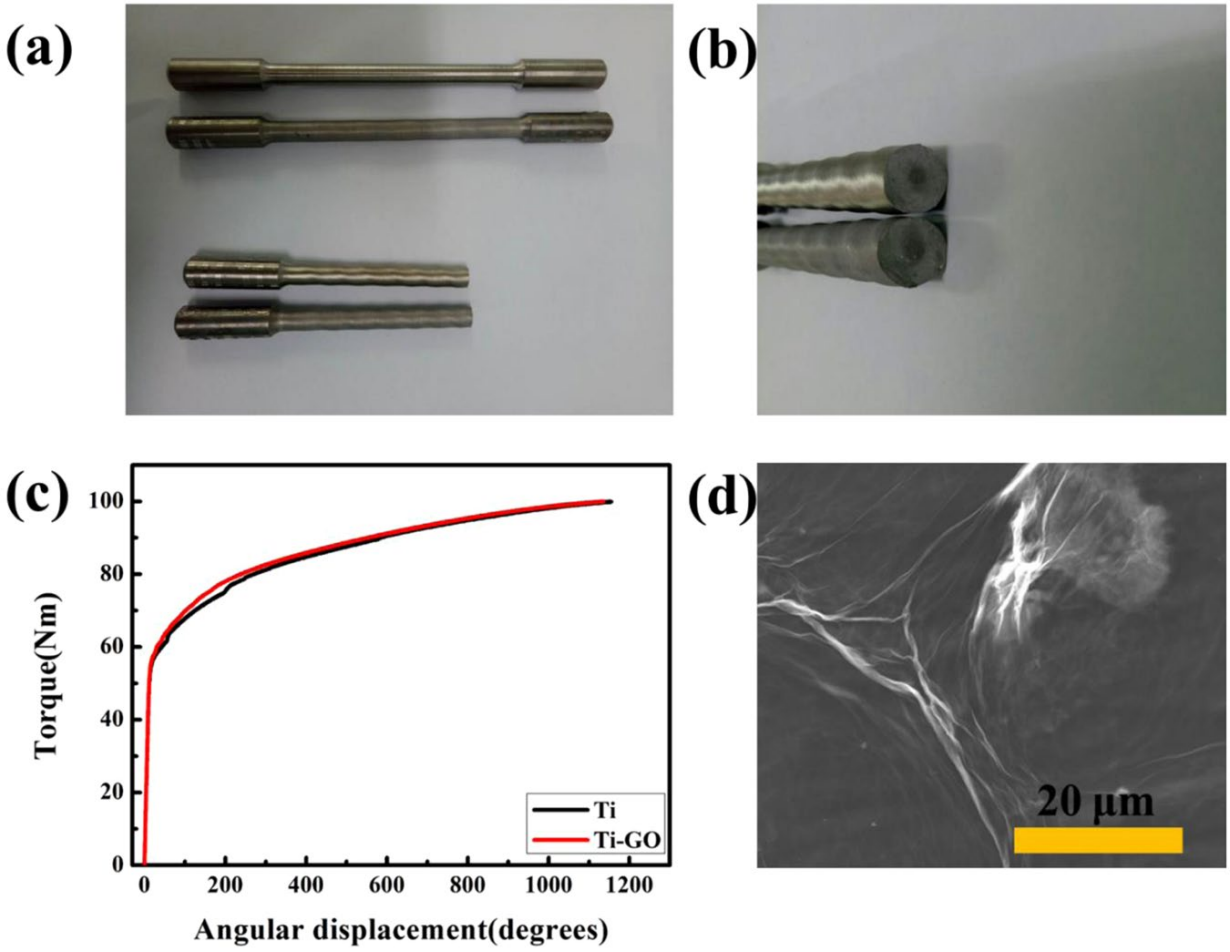
Results of torsion tests. Digital photos: (**a**) Side view of original Ti, original Ti-GO, fractured Ti, and fractured Ti-GO from top to bottom. (**b**) Cross-sectional view of fractured Ti and Ti-GO from top to bottom. (**c**) Plot of torque versus angular displacement. When the torque is less than the elastic limit, linear elastic deformation occurred. (**d**) FE-SEM: A notable and stable GO coating was exhibited on the Ti surface at a torque of 9.48 Nm.

Digital photos (Fig. 6a,b) show that Ti and Ti-GO exhibited shear failure under the action of a torsional shear force, and the cross-section was neat. Moreover, the surface of the standard torsion test section becomes threaded by torsion, and the thread texture of Ti is further. The torsion loading process of plastic materials involves four stages: elastic deformation, yield, strengthening, and fracture^38^. Table S1 (online only) shows that for Ti and Ti-GO, the mean elastic limit torque was 58.24 Nm and 58.37 Nm, respectively. The mean relative angle was 25.92° and 25.38°, respectively. A plot of torque versus angular displacement is shown in Fig. 6c. It reveals a sharp beginning with an almost linearly increasing torque up to a value at which the torque is less than the elastic limit. After the initial stage, there is a plateau region with slowly increasing torque. Remarkably, the microstructure exhibited a notable and stable GO coating on the Ti surface at a torque of 9.48 Nm (Fig. 6d).

### Drug release from Ti and Ti-GO in PBS

GO, which has a large surface area, possesses many π-conjugated structures in the basal layer, providing many opportunities to form strong π-π stacking interactions with π-conjugated systems such as benzene ring in drugs^39^. Aspirin, a model drug, was loaded onto the surface of Ti-GO via simple mixture and π-π stacking interactions between GO and aspirin. The amount of aspirin loaded onto Ti and Ti-GO was around 0.060 mg and 0.076 mg, respectively, as measured by UV–Vis spectra.

To reveal the potential use of GO coating as a carrier for the controlled release of aspirin, we compared the drug release profiles for Ti and Ti-GO. The drug release profiles (Supplementary Fig. S3b) show that the drugs were released directly from Ti very quickly in PBS at 37 °C. After the initial 30 min, about 70.56% of the drugs were released from Ti. After immersion for 2 h, the cumulative release rate from Ti reached 81.67% and after 6 h, 92.22% was released. However, when loading onto Ti-GO, the aspirin-release rates dramatically decreased. Only 33.06% was released after 30 min, and 46.49% was released after 2 h. After immersion for 72 h, 92.1% of the aspirin was released from Ti-GO.

### MC3T3-E1 cell proliferation and adhesion with direct incubation on various surfaces

**Figure 7:**
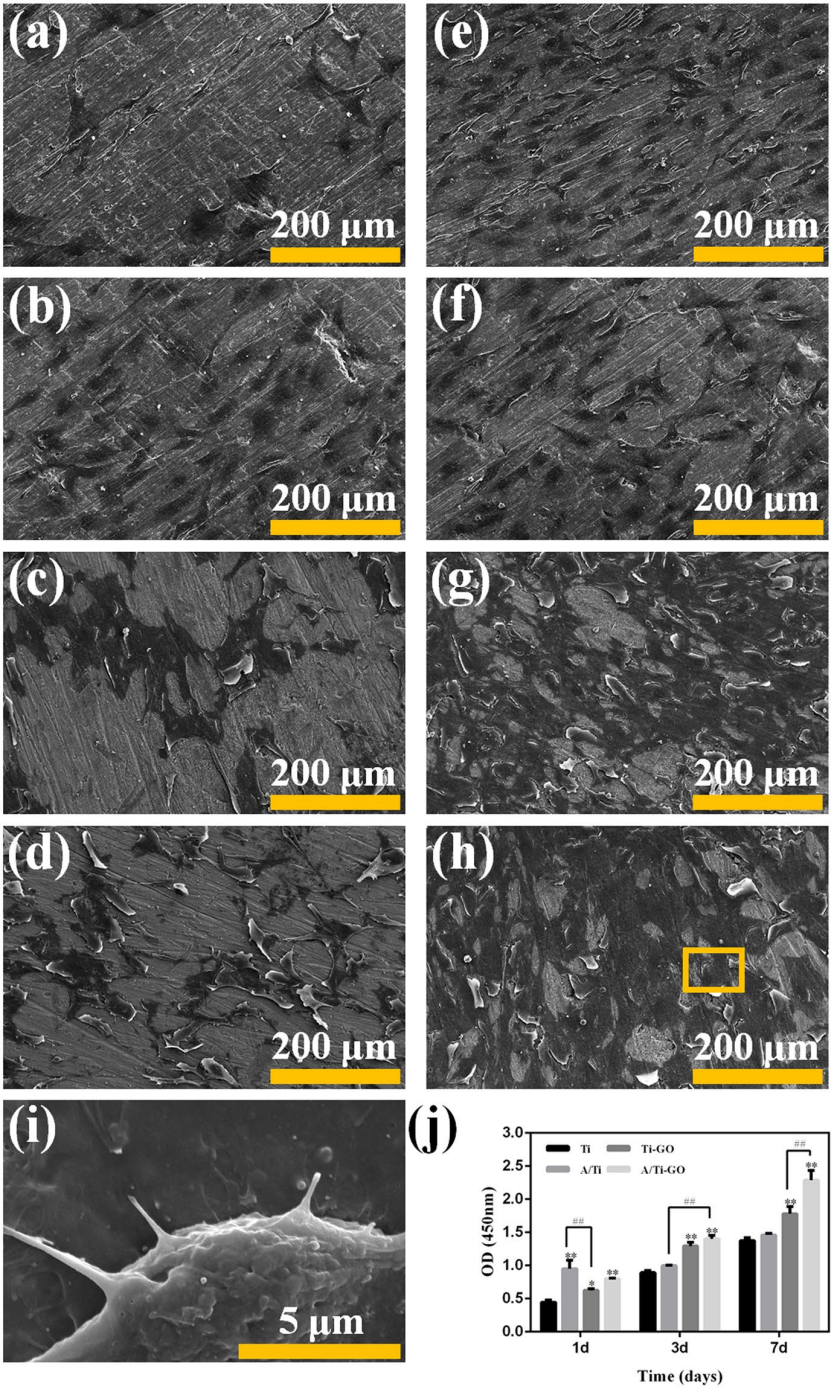
MC3T3-E1 cell attachment and proliferation. FE-SEM of MC3T3-E1 cells cultured on (**a**) Ti, (**b**) A/Ti, (**c**) Ti-GO, and (**d**) A/Ti-GO surfaces on day 1 and (**e**) Ti, (**f**) A/Ti, (**g**) Ti-GO, and (**h**) A/Ti-GO surfaces on day 3, (**i**) higher magnification of the yellow square in (**h**). (**j**) MC3T3-E1 cell proliferation on Ti, A/Ti, Ti-GO, and A/Ti-GO surfaces after culture on days 1, 3, and 7. (Data are presented as mean ± SD, *p < 0.05, **p < 0.01, vs. Ti, ^##^*p* < 0.01, n = 3).

Early cell–biomaterial interaction strongly affects cell behaviour in terms of cell morphology and spreading, which are critical determinants of the cell proliferation rate and differentiation^40,41^. Thus, we evaluated MC3T3-E1 cell proliferation on different surfaces on days 1, 3, and 7 by WST-8 assay (Fig. 7j). After day 1 of culture, the proliferation of MC3T3-E1 cells on both A/Ti and A/Ti-GO was significantly enhanced compared with that on Ti (*P* < 0.01). Interestingly, after days 3 and 7 of culture, A/Ti-GO had the most cells among all groups. All these results suggest that MC3T3-E1 cells proliferated well on these substrates with no apparent toxicity, which can be explained by the stability of the GO coating structure and aspirin release.

The cell morphology results revealed the same trend as the cell proliferation results (Fig. 7a–h). After day 1 of culture, the MC3T3-E1 cells on all the samples had elongated morphology, especially those on the A/Ti-GO samples. There were more cells on the A/Ti sample than on the others. In addition, the number of cells on all the samples increased from days 1 to 3 and the MC3T3-E1 cells maintained the spindle-like elongated morphology, suggesting that they were metabolically active on all the surfaces. In particular, the cells covered the whole surface, presenting a complex layer of growth. Furthermore, multi-branched filopodial protruding extensions and a large number of MC3T3-E1 cells that secreted granular stroma on the A/Ti-GO samples could be observed in the higher magnification micrographs (Fig. 7i).

### Osteogenic differentiation

**Figure 8:**
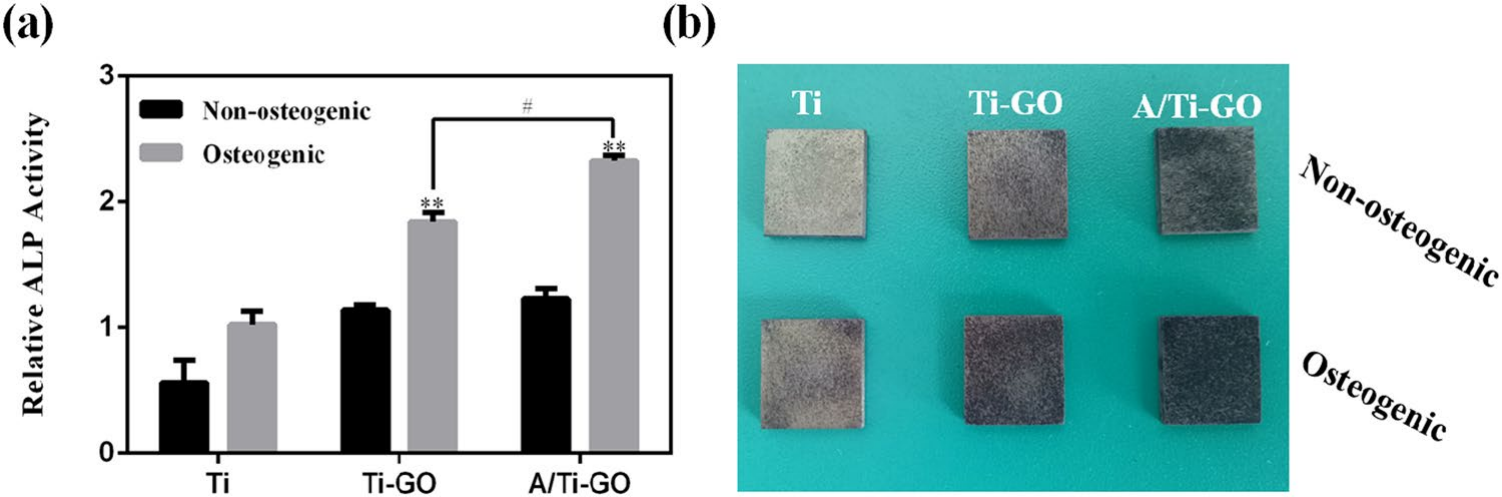
Osteogenic differentiation of MC3T3-E1 cells on Ti, Ti-GO, and A/Ti-GO. (**a**) ALP activities of cells cultured in nonosteogenic and osteogenic supplement media for 7 days. (Data are presented as mean ± SD, **p < 0.01, vs. Ti, ^#^*p* < 0.05, n = 3). (**b**) Digital photos of ALP staining.

It is important to facilitate the differentiation of stem cells into osteoblasts on the surface of the implant for osseointegration after implantation surgery. ALP expression is the most common early marker used for osteogenic differentiation. Figure 8a shows ALP expression by MC3T3-E1 cells on Ti, Ti-GO, and A/Ti-GO. MC3T3-E1 cells cultured in osteogenic medium showed higher ALP expression than cells cultured in non-osteogenic media. The cells on A/Ti-GO showed the highest ALP activity among all the other groups. As expected, ALP staining showed the same results (Fig. 8b). The staining colour and area were most noticeable on A/Ti-GO followed by Ti-GO in osteogenic medium, and the lightest staining was seen on Ti in non-osteogenic medium. Taken together, A/Ti-GO surfaces were most effective in inducing MC3T3-E1 cells to express ALP in the early stages.

## Discussion

In this study, we explored the use of GO coating as a vector for loading aspirin onto the surface of Ti. Among the previously used modification procedures, silanation modification has been used to produce active groups for covalently conjugating GO. The conjugated structure of the silane coupling agent could interact with the hydroxyl group in Ti-NaOH. In addition, the silane coupling agent with amino groups could adsorb the O-containing functional groups of GO. Hence, the ultrathin GO film was successfully conjugated onto the Ti surfaces.

Ti surface hydrophilicity is a key factor that regulates osteogenic cell responses during dental implant healing. A previous report revealed the strong promotion of early osteogenic cell adhesion and maturation by a hydrophilic surface^42^. Because of the formation of a nanogrid structure and the active hydroxyl groups, the surface hydrophilicity for the Ti-NaOH samples was significantly improved. However, Ti-APTES exhibited a slight increase in the contact angle due to the hydrophobic groups such as surface amino groups. The GO film coating increased the content of hydrophilic polar functional groups, as indicated by the XPS analysis in Fig. 5a, and this resulted in decreased contact angle. Consequently, it appeared that the modified Ti-GO possessed extremely good biocompatibility^43^. Hydrophilic surfaces exhibit higher levels of protein adsorption, which promotes cell adhesion and rapid osseointegration^44^.

Cytotoxicity evaluation is a critical step in a cytocompatibility evaluation of a new biomaterial. The WST-8 assay is commonly used for cytotoxicity testing, as it is sensitive and reproducible. The results indicated that Ti-GO was nontoxic to MC3T3-E1 cells. Compared with the Ti extract (Fig. S2), the Ti-GO extract (at a concentration of 100%) showed increased promotion of cell proliferation, which might be attributable to improved MC3T3-E1 cell proliferation due to the GO coating^45^.

Bonding strength is an important factor in the successful implantation of implants with artificial coating materials. The stretch test is a common technique used to evaluate the adhesion of a thin coating on the substrate required for biomedical applications^46,47^. However, this test could not be used to detect the bonding strength between Ti and GO, because the GO coating was ultrathin. In a clinical setting, a dental implant would undergo placement involving rotation as a result of the action of a torque wrench. Therefore, in this study, the torsion test (which was accepted as an important test for implant osseointegration research and immediate loading in previous experiments^48,49^) was conducted to assess the bonding strength between the coating and substrate, simulating the clinical rotating implantation process.

Figure 6b shows that the cross-sections of Ti and Ti-GO are neat, which is in accordance with the conclusion that Ti is a plastic material with high tensile strength, weak shear resistance, and good plastic deformation^50–52^. On the contrary, brittle metal materials such as cast iron tend to be destroyed in the main stress direction (which is at about 45° to the axis) and a threaded surface is not formed. In other words, owing to the weak tensile strength of a brittle material, the fracture will occur along the maximum tensile stress surface^53^. Torsional shear stress can be calculated according to the formulae τ = T/W_P_ and W_P_ = πd^3^/16, where τ is the torsional shear stress, T is the torque, W_P_ is the modulus of torsion resistance, and d is the diameter. In the test, when the torque was 58.37 Nm, the torsional shear stress τ was 580.91 Mpa according to the formula. As is widely known, the torque of an implant placement in the clinical setting is about 30–40 Ncm. If the torque is 50 Ncm and the diameter is 3 mm in the clinical setting, the calculated τ is 94.36 MPa. If the torque increases to 100 Ncm, the shear stress increases to 188 MPa. All the above values are less than the torsional shear stress of 580.91 MPa. In addition, the shear stress decreases as the diameter of the implant increases.

It was found that there were no adverse effects on the normal mechanical properties of pure Ti after GO coating, and the torsional shear stress during implant placement in a clinical setting was within the elastic phase of the material. The abovementioned formulae show that the torque is proportional to the cube of the diameter when the torsional shear stress is the same. When the torque of the implant placement is 50 Ncm, the diameter is 3 mm, the experimental specimen torsion test section diameter is 8 mm, and under the same torsional shear stress, it can be deduced that the torque would be 9.48 Nm. At this torque, FE-SEM showed a stable GO coating on the surface of Ti. The torsion test with Ti-GO revealed that the GO maintained stable bonding, which satisfies the clinical requirements. In subsequent experiments, we will further study the bonding strength between Ti and GO, by simulating implants with threaded structural parts and hollow upper parts connected to the base.

Regarding the drug release from A/Ti and A/Ti-GO in PBS, the initial burst of aspirin release from the surface of Ti happened in the first 0.5 h, but this did not occur for Ti-GO. Because of the interaction between the aspirin benzene ring and functional groups on Ti-GO^25^ (which controlled the aspirin release), the duration of aspirin release was more than 72 h. This indicates that a GO coating on a Ti substrate is a promising approach for drug delivery. We can expect that it will provide a cost-effective method of implant material modification that will be highly useful in the early stage after implantation.

The biological behaviours of cells are highly regulated by the local chemical and topographical environment at the mesoscale, microscale, and nanoscale levels^54–56^. Previous research has demonstrated that TiO_2_ nanotubes with different diameters affect the proliferation and differentiation of mesenchymal stem cells^41^. In this study, the cell proliferation results indicated that cells cultured on A/Ti surfaces proliferated rapidly by day 1, while more cells were observed on A/Ti-GO by days 3 and 7. ALP activity and staining results revealed that cells on A/Ti-GO showed better ability of osteogenic differentiation than those on Ti and Ti-GO. It was probably due to the variation in the duration of aspirin release between A/Ti and A/Ti-GO, indicating that GO coating with loaded aspirin significantly promoted cell proliferation and differentiation. Apart from the drug factor, the alkali-hydrothermal reaction that occurred at the surface of Ti provided a nanotopographic environment (Fig. 3c), which played an important role in controlling stem cell fate on biomaterial surfaces^57^. Furthermore, the hydrophilic environment after GO coating was considerably increased, and cells on hydrophilic surfaces had an elongated morphology in contrast to rounded cells on hydrophobic surfaces^58^. Taken together, GO functionalized with loaded aspirin provided the synergetic effect of chemical cues from the hydrophilic GO and improved proliferation and differentiation induced by aspirin along with topographical cues. Hence, the approach presented here could potentially be used to improve the bioactivity between the Ti implant and surrounding bone tissue. Further longer-term studies are necessary to investigate the osteogenesis potential related to A/Ti-GO *in vitro* and *in vivo*.

## Conclusions

Functional Ti-GO was produced using an alkali-hydrothermal reaction and a coupling agent. Its biocompatibility was then tested using a cytotoxicity test, which indicated that it is safe for clinical applications after the Ti modification. Within the limitations of the present study, the torsion test (simulating the clinical rotating implantation process) revealed the stable bonding strength of the GO coating-Ti interface at a torque of 9.48 Nm, making Ti-GO useful for future applications. Furthermore, aspirin, which has a benzene ring structure, was successfully loaded onto the GO surface, and its release could be sustained for 3 days. *In vitro* cell studies revealed that A/Ti-GO promoted MC3T3-E1 cell proliferation and osteogenic differentiation. Collectively, the results demonstrated that surface-functionalized Ti-GO and additional aspirin loading might be beneficial for further clinical applications in patients with conditions that affect the bone, such as diabetes and osteoporosis.

## Methods

### Synthesis of GO and Ti-GO

The synthesis involved the following steps. First, GO was prepared from natural flake graphite according to a modified Hummers’ method^59^.

Second, the polished Ti samples were immersed in 20 mL of 10 mol/L NaOH aqueous solution, followed by hydrothermal treatment at 60 °C for 24 h. Next, the samples were rinsed repeatedly with ultrapure water and dried at room temperature to obtain the “Ti-NaOH” samples. Subsequently, the Ti-NaOH was immersed in a 3% 3-APTES and ethanol solution for 30 min to introduce positive amine groups onto the Ti surface. The sample was then washed with ethanol and deionized water to obtain Ti-APTES. Afterwards, the sample was gently and thoroughly immersed in 1.0 mg/mL, 1.5 mg/mL, or 2.0 mg/mL GO ultrapure water solution for 1 h, and washed with fresh distilled water and dried at 37 °C to acquire the final GO-coated Ti products (Ti-GO products).

### Characterization methods

The morphology of GO was characterized using tapping mode atomic force microscopy (AFM; Dimension FastScan, Bruker, Madison, California, USA) and transmission electron microscopy (TEM; JEOL JEM-2100, Akishima-shi Tokyo, Japan) by depositing the GO suspension onto mica and copper grid substrates, respectively. The Ti-GO microstructure was studied using field-emission scanning electron microscopy (FE-SEM; FEI Quanta 200, Eindhoven, Netherlands).

X-ray photoelectron spectrometry (XPS; 250Xi, Thermo Fisher, Shanghai, China) was utilized with Al Kα excitation radiation to detect the chemical compositions of the samples. All the XPS spectra were calibrated using the C1s line at 284.8 eV.

In addition, the surface hydrophobicity of the samples was determined using a JCY-2 contact angle analyser (Shanghai FangRui Instrument Co., Ltd, Shanghai, China).

### Cytotoxicity

Mouse osteoblastic MC3T3-E1 cells (Saibaikang Biotechnology Co., Ltd., Shanghai, China) were used to evaluate the cytotoxicity of the samples. The cells were cultured in a humidified atmosphere at 5% CO_2_ and 37 °C using Dulbecco’s modified Eagle’s medium (DMEM; Hyclone, Logan, UT, USA) with 10% foetal bovine serum (FBS; Hyclone) and 1% penicillin/streptomycin (P/S) mixture (ScienCell, California, USA). Before incubation, the samples were sterilized by autoclaving and ultraviolet (UV) irradiation on a clean bench for 2 h. After that, the samples were incubated in DMEM medium (supplemented with 10% FBS and 1% P/S) in a humidified atmosphere at 5% CO_2_ and 37 °C for 72 h. The ratio of sample area to extraction medium was 3 cm^2^/mL. The extract was classed as 100% and diluted to 75% and 50% for further utilization.

Meanwhile, 100 μL of the cell suspension with a cell density of 5 × 10^4^ cells/mL was added to each well of a 96-well culture plate. After culturing for 24 h, the culture medium was replaced with 200 μL of the extract at 100%, 75%, or 50%. Culture medium without extract served as the negative control.

Cell viability was assessed after days 1 and 3 of culture by using a cell counting kit-8 (CCK-8; Dojindo, Japan) with WST-8 (2-(2-methoxy-4-nitrophenyl)-3-(4-nitrophenyl)-5-(2,4-disulfophenyl)−2H-tetrazolium) in accordance with the manufacturer’s instructions. The previous culture medium was replaced with 110-μL fresh DMEM medium containing 10 μL CCK-8. After being incubated for another 4 h, the optical density was assessed using a microplate reader (Thermo Fisher Scientific, Shanghai, China) at 450 nm absorbance, and the cell viability was calculated as follows:

$${\rm{Cell}}\,{\rm{viability}}\,( \% )=({{\rm{A}}}_{{\rm{sample}}}-{{\rm{A}}}_{{\rm{blank}}}/{{\rm{A}}}_{{\rm{control}}}-{{\rm{A}}}_{{\rm{blank}}})\times 100 \% $$

### Torsion test to evaluate bonding strength of Ti-GO

Ti specimens of the standard torsion test section (length × diameter = 125 mm × 8 mm) and standard fixed part (length × diameter = 40 mm × 10 mm) were prepared and coated with GO as described above (Fig. 6a). Briefly, each cylindrical specimen was immersed in 10 mol/L NaOH aqueous solution at 60 °C for 24 h, and then treated with 3 wt% APTES solution for 30 min. After that, the specimen was immersed in 1.5 mg/mL GO solution for 1 h to obtain a GO-coated cylindrical specimen (Ti-GO). The torsion tests were conducted in a 1000 Nm capacity test machine (NDW 31000; KEXIN Inc., Changchun, China). The torque was applied at a rate of 20°/min, and the torque and angle of rotation were recorded by the sensors of the test machine. To obtain reliable plots of torque versus angle of rotation, three specimens were used for each torsion test. The stability of the GO coating on a cylindrical Ti-GO specimen was analysed by FE-SEM with a torque of 9.48 Nm.

### *In vitro* drug release test

To determine the maximum absorption wavelength of aspirin, a mixture of 0.5 mg/mL aspirin in ethanol was assessed based on UV-Vis spectrometer measurements, using a NanoDrop-2000 (Thermo Scientific Corporation, Massachusetts, USA). The results indicated that aspirin displayed characteristic absorption at 276 nm (Fig. S3a).

Aspirin was employed as a model drug, and it was loaded onto the Ti and Ti-GO samples according to a previous literature report^60^. Simply, aspirin (50 mg) dissolved in ethanol (100 mL) was sonicated for a few minutes to mix it sufficiently and then a 20 μL aspirin solution was dropped onto the Ti and Ti-GO samples, followed by drying in air at room temperature. This procedure was repeated 10 times. Unabsorbed aspirin was removed by washing thrice with phosphate-buffered saline solution (PBS) to obtain the aspirin-loaded A/Ti and A/Ti-GO samples. The extent of aspirin loading onto Ti and Ti-GO was determined with the UV–Vis spectrometer by measuring the concentration of aspirin in the upper layer after sonication for 3 h. The assessment was based on the aspirin standard curve obtained at a wavelength of 276 nm from a series of aspirin solutions at different concentrations.

The aspirin-release behaviour was assessed using A/Ti and A/Ti-GO samples immersed in 1 mL PBS at 37 °C for 3 d. After incubation for 30 min, 2 h, 6 h, 24 h, 48 h, and 72 h, 10 μL PBS was collected and replaced with 10 μL fresh PBS. The amount of released aspirin was determined based on the aspirin standard curve at 276 nm, and the curve (over time) of the cumulative mass of released aspirin was plotted. Three A/Ti and A/Ti-GO samples were assessed for each incubation duration for the statistical analysis.

### MC3T3-E1 cell proliferation and adhesion with direct incubation on various surfaces

Before evaluating MC3T3-E1 cell proliferation and adhesion, the Ti, A/Ti, Ti-GO, and A/Ti-GO samples were sterilized with UV irradiation for 2 h. MC3T3-E1 cells were seeded onto the samples in a 24-well plate at a density of 5 × 10^4^ cells/mL. After days 1, 3, and 7 of direct incubation, the MC3T3-E1 cell proliferation on the various samples was measured using a WST-8 assay as described above. The previous culture medium was replaced with 1100 μL fresh DMEM medium containing 100 μL CCK-8. After being incubated for another 4 h, 110 μL of solution from each sample was transferred to a 96-well culture plate, and the absorbance at 450 nm was measured.

The morphologies of the MC3T3-E1 cells on the samples were observed by FE-SEM after days 1 and 3 of culture. First, the samples were fixed in 2.5 v% glutaraldehyde at 4°C in the dark overnight, and then washed twice for 10 min with PBS (pH 7.4). Next, they were dehydrated in 1% OsO_4_ for 1 h and gently rinsed again with PBS. The cells were then immersed in 75% and then 90% (v/v) ethanol/water solutions (15 min each) at 4°C, followed by immersion in 100% ethanol (3 × 10 min) at room temperature. The morphologies of the MC3T3-E1 cell on the samples were observed by FE-SEM after drying with tertiary butyl alcohol and sputtering with gold.

### Osteogenic differentiation

Osteogenic differentiation of MC3T3-E1 cells was studied by measuring the alkaline phosphatase (ALP) activity and ALP staining in DMEM medium (supplemented with 10% FBS and 1% P/S) and in a medium supplemented with osteoinductive factors (10^−8^ mol/L dexamethasone, 10 mmol/L β-glycerophosphate and 50 µg/mL ascorbic acid; all purchased from Sigma).

ALP expression was measured using the ALP activity assay kit (JianCheng Biotech, Nanjing, China) in accordance with the manufacturer’s instructions. For the analysis of ALP activity, cell lysates were isolated using a cell lysis buffer on day 7, and ALP substrate buffer containing 5 mmol/L of p-nitrophenol (pNP) was added to each group. After incubation at 37 °C for 15 min, the absorbance at 520 nm was measured with a microplate reader. The ALP activity value was normalized by protein content measured at 562 nm.

The ALP staining process was as follows. First, the samples were fixed in 4% (w/v) paraformaldehyde for 30 min, and then dye solution was added and the samples incubated in the dark for 1 h. In the final step, the dyeing conditions of the MC3T3-E1 cells on the samples were recorded by a digital camera.

### Statistical analysis

The data are expressed as the means ± SD, and all experiments were repeated at least three times. Statistically significant differences between the samples were analysed using one-way analysis of variance (ANOVA) with Tukey’s test for multiple comparisons, and *P* < 0.05 was considered to represent statistical significance. All statistical procedures were carried out using GraphPad Prism 5 statistical software (GraphPad, California, USA).

## Electronic supplementary material

Supplementary Information
