# Supplementary material for: Effects of aspirin-loaded graphene oxide coating of a titanium surface on proliferation and osteogenic differentiation of MC3T3-E1 cells
Source: Sci Rep. 2018 Oct 11;8:15143. doi: 10.1038/s41598-018-33353-7 (PMC6181949; doi:10.1038/s41598-018-33353-7)
Supplement: Supplementary file 1 — Supplementary Information [file 41598_2018_33353_MOESM1_ESM.doc]

Supplementary Information

**Effects of aspirin-loaded graphene oxide coating of a titanium surface on proliferation and osteogenic differentiation of MC3T3-E1 cells**

Liping Ren^1^, Shuang Pan^2,4^, Haiqing Li^3^, Yanping Li^2^, Lina He^2^, Shuang Zhang^2^, Jingyi Che^2^ , Yumei Niu^2,4,*^.

^1^Department of Prosthodontics, The First Affiliated Hospital of Harbin Medical University, No. 143 Yiman Street, Nangang District, Harbin 150001, China.

^2^Department of Endodontics, The First Affiliated Hospital of Harbin Medical University, No. 143 Yiman Street, Nangang District, Harbin 150001, China.

^3^Department of Stomatology, Hospital of Heilongjiang Province, No. 82 Zhongshan Street, Xiangfang District, Harbin 150036, China.

^4^Oral Biomedical Research institute of Harbin Medical University, No. 143 Yiman Street, Nangang District, Harbin 150001, China.

^*^Correspondence: Yumei Niu, Department of Endodontics, The First Affiliated Hospital of Harbin Medical University. Oral Biomedical Research institute of Harbin Medical University, No. 143 Yiman Street, Nangang District, Harbin 150001, China. Tel: 86-451-85553234, Fax: 86-451-53625108. [yumeiniu@163.com](mailto:yumeiniu@163.com)

**Materials.**

Pure Ti (10 mm × 10 mm × 1 mm) obtained from Northeast Light Alloy Co. Ltd. (Harbin, China) was mechanically polished using wet sandpaper with a grit size up to #1200; ultrasonically cleaned with acetone, ethanol, and ultrapure water successively (each for 10 min); and readied for subsequent experiments. Pyrrole, phosphoric acid (H_3_PO_4_), hydrogen peroxide (H_2_O_2_), sulphuric acid (H_2_SO_4_), potassium permanganate (KMnO_4_), and sodium hydroxide (NaOH) were supplied by Sinopharm Chemical Reagent Company (Shanghai, China), and used without further purification. Natural flake graphite, with a mean particle diameter of 200 mesh, was purchased from Nanjing XFNANO Material Tech. Co., Ltd. (Nanjing, China). 3-aminopropyltrienthoxysilane (3-APTES) dissolved in ethanol to obtain a 3wt% APTES and ethanol solution, and aspirin, were purchased from Dalian Meilun Biological Technology Co., Ltd. (Dalian, China).

**Synthesis of GO**

Briefly, sulphuric acid was mixed with phosphoric acid in a ratio of 9:1. Next, a moderate amount of natural flake graphite was added and vigorously stirred in at 5℃. KMnO_4_ was added and stirred in for 30 min. After the addition of the oxidant, the beaker was heated and kept at 50℃ with continuous stirring for 10 h. It was then left at room temperature overnight. After the completion of this reaction, the reddish-brown sticky mixture was diluted with deionized water and the unreacted KMnO_4_ was removed by H_2_O_2_. In the next step, the mixture was rinsed with HCl solution to remove the sulphate ions and washed repeatedly with deionized water until the pH of the supernatant was neutral to remove the chloride ions. Subsequently, a brown powder was obtained after drying with P_2_O_5_ in a desiccator. The powder was then dispersed in ultrapure water by 30 min of ultrasonication to produce an aqueous dispersion of GO.


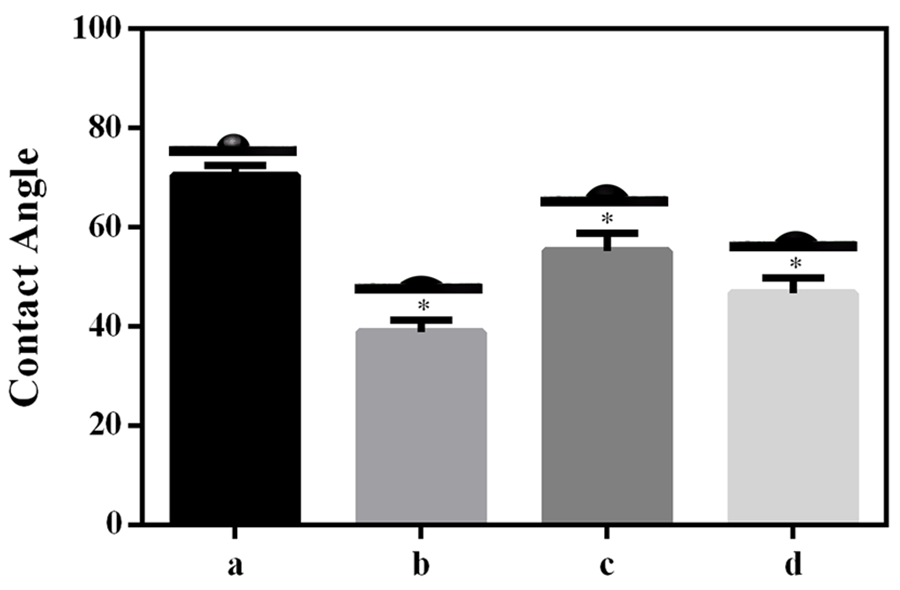


**Figure S1. Water contact angle** (**a**) Ti, (**b**) Ti-NaOH, (**c**) Ti-APTES, (**d**) Ti-GO. (All data are presented as mean ± SD, **p*<0.05, n=6, vs. Ti.)


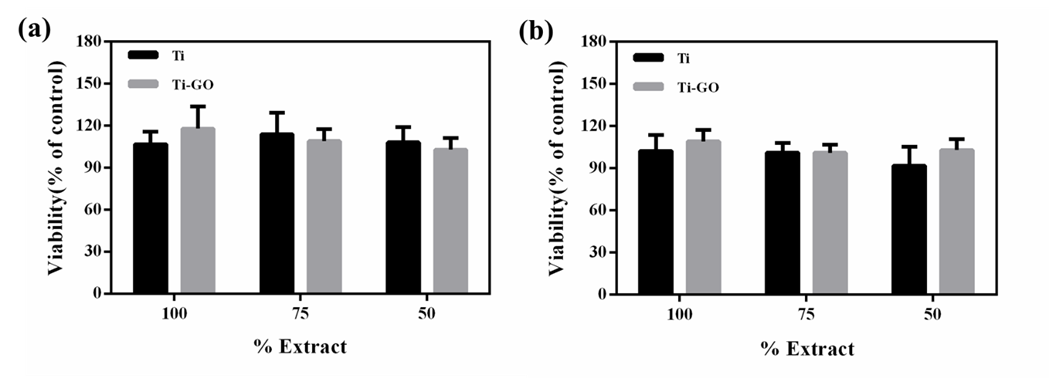


**Figure S2. Viability of MC3T3-E1 cells incubated with different concentrations of Ti and Ti-GO extracts (100%, 75%, and 50%).** No significant difference in cell proliferation was observed with respect to (**a**) 1-day and (**b**) 3-day cultures between Ti and Ti-GO, indicating that Ti-GO was nontoxic to MC3T3-E1 cells. All data are presented as mean ± SD. (*p*>0.05, n=6.)

**Table-S1. Results of torque test for line elastic limit value (mean ± SD, n=3).**

| Sample Ti Ti-GO  Angle (°) Torque (Nm) Angle (°) Torque (Nm) |
| --- |
| 1 28.52 58.73 22.54 56.65  2 22.52 56.78 20.25 57.90  3 26.71 59.23 33.34 60.50  Mean (SD) 25.92 (2.513) 58.24 (1.066) 25.38 (5.708) 58.37 (1.626) |


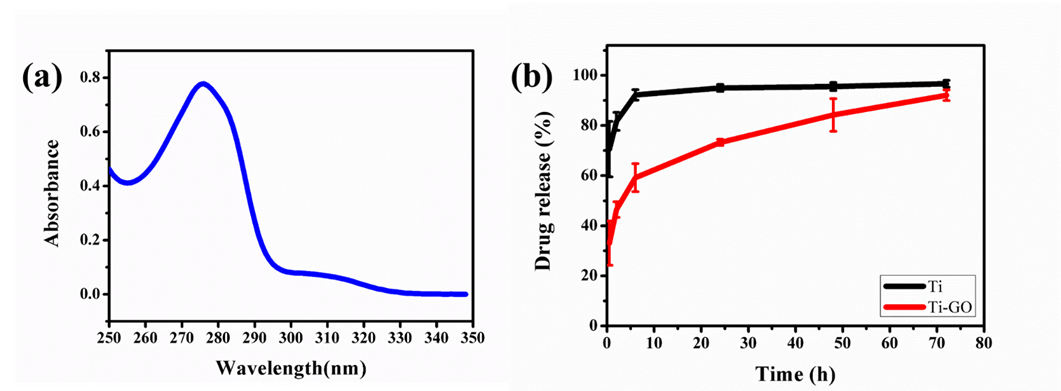


**Figure S3. Aspirin delivery** (**a**) UV-vis absorbance of aspirin. (**b**) Cumulative release profiles of Ti and Ti-GO samples in PBS at 37℃ (measured by UV-vis spectrometry) over 3 days. (mean ± SD, n=3).
